# Highly Selective Electrochemical Reduction of CO_2_ into Methane on Nanotwinned Cu

**DOI:** 10.1021/jacs.3c00847

**Published:** 2023-04-18

**Authors:** Jin Cai, Qing Zhao, Wei-You Hsu, Chungseok Choi, Yang Liu, John Mark P. Martirez, Chih Chen, Jin Huang, Emily A. Carter, Yu Huang

**Affiliations:** † Department of Materials Science and Engineering, University of California, Los Angeles, Los Angeles, California 90095-1595, United States; ‡ Department of Mechanical and Aerospace Engineering and the Andlinger Center for Energy and the Environment, Princeton University, Princeton, New Jersey 08544-5263, United States; § Department of Materials Science and Engineering, National Yang Ming Chiao Tung University, Hsinchu 30010, Taiwan, ROC; ∥ Department of Chemical and Biomolecular Engineering, University of California, Los Angeles, Los Angeles, California 90095-1592, United States; ⊥ California NanoSystems Institute, University of California, Los Angeles, Los Angeles, California 90095, United States

## Abstract

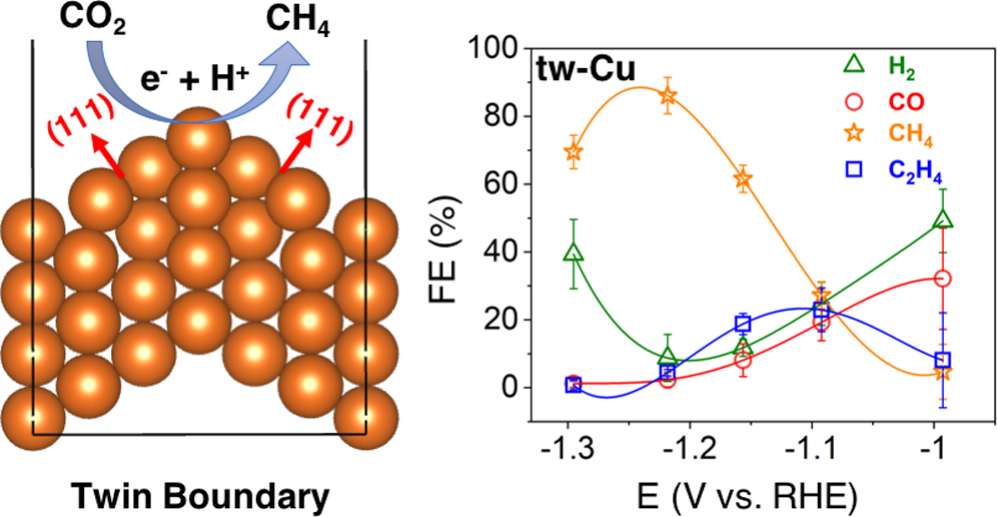

The electrochemical carbon dioxide reduction reaction (CO_2_RR) is a promising route to close the carbon cycle by reducing CO_2_ into valuable fuels and chemicals. Electrocatalysts with high selectivity toward a single product are economically desirable yet challenging to achieve. Herein, we demonstrated a highly (111)-oriented Cu foil electrocatalyst with dense twin boundaries (TB) (tw-Cu) that showed a high Faradaic efficiency of 86.1 ± 5.3% toward CH_4_ at −1.2 ± 0.02 V vs the reversible hydrogen electrode. Theoretical studies suggested that tw-Cu can significantly lower the reduction barrier for the rate-determining hydrogenation of CO compared to planar Cu(111) under working conditions, which suppressed the competing C–C coupling, leading to the experimentally observed high CH_4_ selectivity.

## Introduction

The electrochemical reduction of carbon dioxide (CO_2_) offers a promising means for storing intermittent renewable energy in chemical fuels, promoting the usage of carbon-neutral energy in transportation and chemical sectors.^1−4^ To date, copper (Cu) remains the most effective electrocatalyst for the CO_2_ reduction reaction (CO_2_RR) for producing hydrocarbons and oxygenates.^5^ A variety of molecular products containing one to three C atoms (C_1_–C_3_) have been reported on Cu-catalyzed CO_2_RR,^5−7^ which shows great economic potential for producing chemical fuels using CO_2_.^8^ Among these hydrocarbon products, methane (CH_4_), the simplest of them, is of particular interest due to its good compatibility with existing natural gas infrastructure.^9^ CH_4_ synthesized from CO_2_RR is a carbon-neutral alternative to extracted natural gas. There is a large global demand for methane, while the production of CH_4_ in the U.S. is heavily dependent on the fracking of shale, which, if unregulated and not done properly, can inflict irreversible damage on the environment.^9^ Displacing conventional CH_4_ production with electrochemical CO_2_RR potentially can contribute to a net zero CO_2_ emissions economy if excess renewable or nuclear power is used, which is a key motivation for electrochemical CO_2_RR. In addition, utilization of CH_4_ leverages the well-established infrastructure to store, transport, and use methane as a fuel.^10^ However, current Cu-based catalysts exhibit insufficient CH_4_ selectivity, resulting in a high economic penalty in post-reaction separation, contributing to the current limited deployment of electrochemical CO_2_RR.^11^

It has been widely acknowledged that the CO_2_RR pathways are highly dependent on the catalyst surface structure.^12^ For example, a close-packed Cu(111) surface is more selective toward CH_4_ compared to a more open Cu(100) surface.^13−15^ In addition, defects such as steps and grain boundaries exposed on the surface have been found to influence CO_2_RR and CO reduction selectivity.^16−18^ Grain boundaries (due to their prevalence in polycrystalline Cu, pc-Cu) have been studied extensively for CO_2_RR and the CO reduction reaction (CORR), to understand their roles in CO_2_RR. Feng et al. found that the CORR activity of Cu for reducing CO to C_2_ products was linearly proportional to the density of grain boundaries.^19^ Verdaguer-Casadevall et al. demonstrated that CORR active sites on oxide-derived Cu surfaces were located at grain boundaries, which bind CO strongly.^20^ Chen et al. further confirmed that the enhanced CO binding on grain boundaries can lead to high selectivity to C_2_ product yield in CO_2_RR.^21^ Although studies found that certain active sites in oxide-derived pc-Cu structures may promote the production of C_2_ molecules, grain boundaries usually contain a variety of active sites, which limits their selectivity toward a single product.^22^ In addition, due to the existence of a diversity of sites, the underlying atomic-scale mechanism of CO_2_RR on grain boundaries is not yet well established.

A twin boundary is a special type of boundary interface common in face-centered-cubic (FCC) crystals such as Cu. Twin-boundary energies are usually an order of magnitude smaller than high-angle grain boundaries,^23^ which makes them more stable and amenable to mechanistic studies of structure-sensitive CO_2_RR. Herein, we present a highly (111)-oriented Cu foil electrocatalyst with dense twin boundaries (tw-Cu) with an exceptional CH_4_ selectivity of 86.1 ± 5.3% at −1.2 ± 0.02 V vs the reversible hydrogen electrode (RHE). High-level quantum mechanical simulations show that the incorporation of twin boundaries on Cu(111) facets greatly reduces the reaction barrier of the rate-limiting CO hydrogenation step for CH_4_ formation compared to the planar Cu(111) surface, explaining the high CH_4_ selectivity observed in experiments.

## Experimental Methods

### Chemicals

Ethanol (C_2_H_5_OH) (200 proof) and potassium bicarbonate (KHCO_3_) were purchased from Sigma-Aldrich. Deionized (DI) water (18.2 MΩ cm) produced by an ultrapure purification system (Milli-Q advantage A10) was used to make the solutions. Pc-Cu was purchased from Sigma-Aldrich with 99.999% purity. The Nafion 115 membranes were purchased from the Fuel Cell Store. All reagents were used as received without further purification.

### Synthesis of Tw-Cu

In this study, we adopted rotary electroplating to fabricate tw-Cu foils.^24,25^ The electrolyte contains copper sulfate (CuSO_4_), hydrochloric acid (HCl), sulfuric acid (H_2_SO_4_), and additives for nanotwin growth were provided by Chemleaders, Inc. Figure S1 shows the schematic diagram for the rotary electroplating system, in which the inert anode is titanium (Ti) coated with iridium dioxide (IrO_2_) and the cylinder cathode is made from Ti. During the electroplating process, the cathode rotation speed was 800 rpm controlled by a modulated speed rotator. The current density was 11 ASD (A/dm^2^), and the thickness of tw-Cu foils was about 45 μm. Due to advantageously poor adhesion between the Ti and the tw-Cu foils, the electroplated tw-Cu foils were peeled off after deposition for subsequent studies.

### Structure Characterization

Transmission electron microscopy (TEM) samples were prepared by a Nova 600 SEM/FIB system. TEM images of tw-Cu were taken using an FEI Titan scanning transmission electron microscope (STEM) at an acceleration voltage of 300 kV. Atomic force microscopy (AFM) images of the Cu foils were taken using a Bruker Dimension FastScan Scanning Probe Microscope (SPM) under ScanAsyst mode. Scanning electron microscopy (SEM) images were taken by a ZEISS Supra 40VP SEM. The crystal structure of the Cu foil was analyzed with a Panalytical X’Pert Pro X-ray powder diffractometer (XRD) using a Cu Kα radiation source and conducted with a symmetric scan. Electron backscatter diffraction (EBSD) was measured with a TESCAN GAIA-3 XMH integrated FIB-FESEM.

### Electrochemical Measurement

All electrochemical experiments were conducted in an H-shaped cell (H-cell) composed of two compartments and separated by a proton-exchange membrane. The cell was sonicated with 2% nitric acid and boiled with DI water three times before each test. The tw-Cu and pc-Cu foil were cut to 0.3 cm^2^ and electrochemically polished in 85% phosphoric acid using samples as the anode and a Cu foil as the cathode under 2 V vs Ag/AgCl for 200 s and then rinsed with DI water before each test. The tw-Cu and pc-Cu foils were fixed by an electronic clip to form the working electrode. The counter electrode was a Pt wire from Pine Instruments. A Ag/AgCl (4 M KCl) electrode purchased from Pine Instruments was used as the reference electrode. 0.1 M KHCO_3_ electrolyte was prepared as the electrolyte. A stirring bar was introduced to the cathode chamber to mix the electrolyte thoroughly. A glass gas dispersion purging tube was inserted into the cathode chamber. CO_2_ (Air Gas, 99.99%) was purged at a rate of 11 sccm for 25 min before and during all electrocatalytic measurements. Electrochemical measurements were performed using a Princeton potentiostat (VersaSTAT 4). All current density was normalized by the geometric area. A constant voltage was applied for 20–30 min before the effluent was injected into a gas chromatograph (GC). Electrochemical data were recorded vs the reference electrode and converted to the RHE scale after iR correction.

### Product Analysis

Gas products were analyzed by a GC instrument (Shimadzu GC-2010-Plus) equipped with a Barrier Ionization Discharge (BID) detector and a Restek ShinCarbon ST Micropacked column (2 m × 1 mm ID). Helium ISP (Air Gas, 99.9999%) was applied as the carrier gas. The H-cell was connected to the GC with an outlet gas line. The effluent was injected through a six-port valve with a sampling loop of 1.5 mL effluent gas. The column oven was maintained at 30 °C for 8 min followed by a temperature ramping at 8 °C min^–1^ to 250 °C, which was maintained for 5 min. The external standard method was used for quantitative calculations. A calibration curve was made by analyzing a series of standard gas mixtures (Air Gas), with the concentration of the standard gas as the vertical axis and the respective peak area as the horizontal axis. After the calibration curve was created, the concentration of the sample could be calculated from the calibration curve based on the peak area detected under the same condition.

The Faradaic efficiency (FE) was calculated from
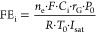
where *n*_e_ is the number of electrons transferred; *F* is the Faraday constant (96 485 C mol^–1^); i is the species, H_2_, CO, CH_4_, or C_2_H_4_; *C*_i_ is the concentration of the gas read from GC-BID; *r*_G_ is the gas flow rate acquired from a ProFlow 6000 electronic flow meter (Restek) at the exit of the electrochemical cell (mL min^–1^ at room temperature and ambient pressure); *P*_0_ is the atmospheric pressure (101 325 Pa); *R* is the ideal gas constant (8.314 J mol^–1^ K^–1^); *T*_0_ is the room temperature (298.15 K); and *I*_sat_ is the current after saturation.

Liquid products were analyzed by quantitative NMR spectroscopy (Bruker AV-300). Specifically, 0.1 mL of D_2_O was added to 0.9 mL of the cathode electrolyte and 10 μL of dimethyl sulfoxide (17.75 μM) was also mixed in as an internal standard. The one-dimensional ^1^H spectrum was measured with a prewater saturation method.

### Computational Details

We performed spin-polarized periodic Kohn–Sham density functional theory (DFT) calculations with the all-electron, frozen-core, projector augmented-wave (PAW)^26^ method, Perdew–Burke–Ernzerhof (PBE) exchange-correlation functional,^27^ and Grimme’s D3 dispersion correction^28,29^ with Becke–Johnson damping^30^ using the Vienna Ab initio Simulation Package (VASP)^31,32^ version 5.4.4. We self-consistently simulated the valence 1s of H, 2s and 2p of C and O, and 4s and 3d of Cu. We employed a four-layer 4 × 6 supercell containing 96 Cu atoms along with at least 15 Å of vacuum to model the tw-Cu(111) surface (Figure 3A). We relaxed the atomic positions in the two topmost Cu layers and fixed the atoms in the two bottommost Cu layers at their bulk atomic positions. We applied dipole-field energy and potential corrections^33^ along the *z*-direction to cancel the artificial electrostatic interaction between the slabs. We used a kinetic energy cutoff of 660 eV for the plane-wave (PW) basis set, along with a Γ-point-centered Monkhorst–Pack^34^*k*-point mesh of 4 × 4 × 1 to sample the Brillouin zone. We used Fermi surface smearing with a width of 0.09 eV within the Methfessel–Paxton scheme^35^ for Brillouin zone integration to aid self-consistent-field convergence. We relaxed all atoms until the absolute total force on each atom was smaller than 0.03 eV/Å in geometry optimizations. We optimized the MEPs using the climbing image nudged elastic band (CI-NEB)^36^ method. Details about CI-NEB calculations, treatment of solvent, free-energy calculations, screening adsorption sites of key intermediates, approximating embedded complete active space second-order perturbation theory (emb-CASPT2) barriers, and determining potential dependence of the free energies can be found in Notes S1–S3.

Further details are available in the Supporting Information.

## Results and Discussion

### Surface Structure Study of Tw-Cu and Pc-Cu Catalysts

We first synthesized tw-Cu catalysts using a previously reported approach,^24,25^ through rotary electroplating in a CuSO_4_, HCl, and H_2_SO_4_ mixed electrolyte with Ti used as the cathode and Ti coated with IrO_2_ as the anode (see Figure S1 for details). The resulting 45-μm-thick electroplated tw-Cu foil was then peeled off for subsequent structural characterization and electrochemical CO_2_RR studies.

**Figure 1:**
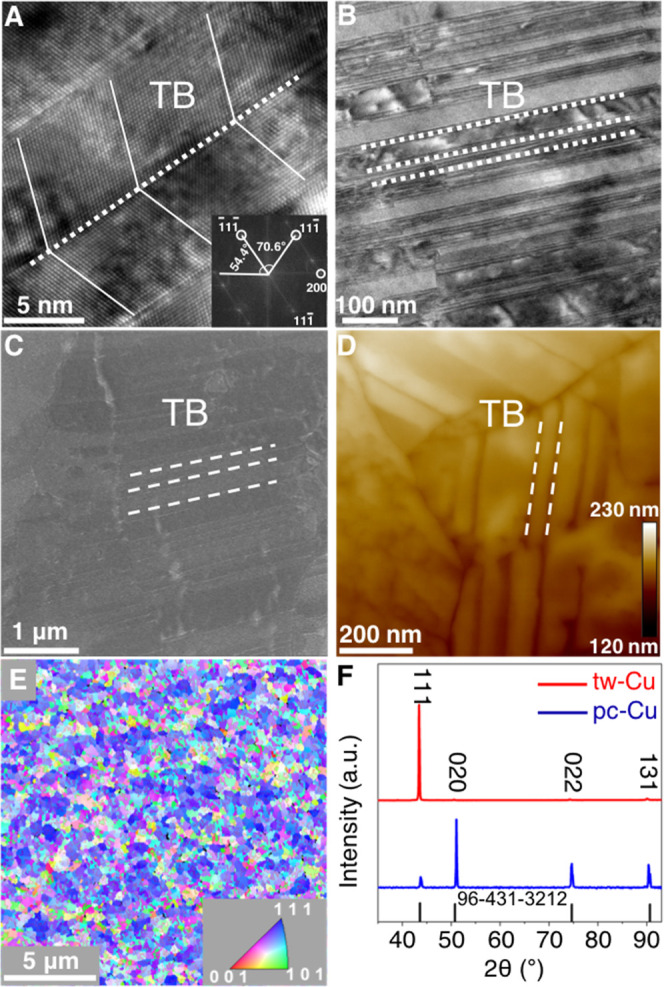
Structural characterization of tw-Cu and pc-Cu catalysts. (A) High-resolution TEM (HRTEM) image of the cross section of tw-Cu with its twin-boundary (TB) assembly. Inset: FFT of the corresponding Cu TEM, which indicates the ⟨110⟩ axial direction and expression of the {111} planes. (B) Low-magnification TEM image of tw-Cu. The white dashed lines mark the typical TB. (C) SEM image of tw-Cu. The white dashed lines mark the TB. (D) AFM image of tw-Cu, which shows a surface roughness (*R*_a_) of 2.7 nm. (E) Plane-view EBSD orientation maps showing the texture of the surface of tw-Cu. The inset indicates the crystallographic vectors used to color orientations in the maps, suggesting a strong (111) texture. (F) XRD pattern of tw-Cu and pc-Cu, showing highly (111)-oriented tw-Cu compared to pc-Cu. The black line represents the reference sample with a PDF number of 96-431-3212.

The cross section of tw-Cu was characterized by TEM, which shows well-defined twin-boundary structures (Figure 1A,B). The stacking sequence is inverted as ABC/A/CBA.^37^ The structure of the tw-Cu was analyzed further by fast Fourier transform (FFT). The inset of Figure 1A shows the FFT with ⟨110⟩ axial direction and expression of the {111} planes. The tw-Cu is composed of nanotwins with an average width of 7 nm, quantified from Figure 1B. SEM and AFM further confirmed the rich twin boundaries on the surface (Figures 1C,D and S2). The twin-boundary density of tw-Cu was determined to be 0.5 μm/μm^2^ by EBSD (Figure 1E), which was much higher than (0.07 μm/μm^2^) that of the commercial pc-Cu foil (99.999% Cu foil, Sigma-Aldrich). The tw-Cu possessed a highly preferred (111)-oriented texture on the surface (Figure 1E), consistent with XRD spectra (Figure 1F).

### Electrochemical CO_2_RR Study

**Figure 2:**
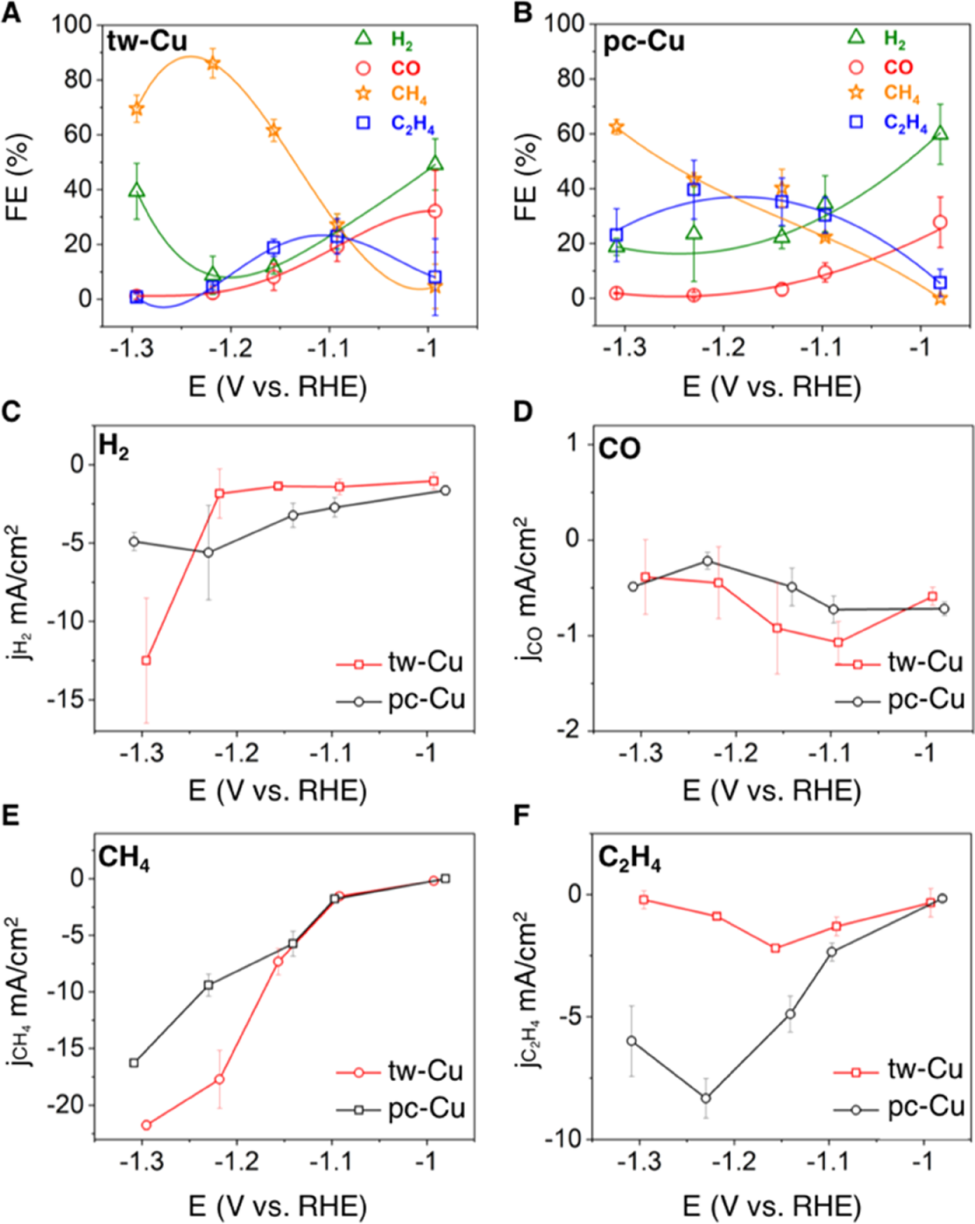
Electrochemical CO_2_RR performance. FEs of (A) tw-Cu and (B) pc-Cu. H_2_, CO, CH_4_, and C_2_H_4_ are denoted, respectively, as green, red, orange, and blue data points. Partial current densities of (C) H_2_, (D) CO, (E) CH_4_, and (F) C_2_H_4_. Red lines represent tw-Cu, and black lines represent pc-Cu. Each error bar was calculated from three independent measurements. All potentials were iR-corrected.

The CO_2_RR performance of tw-Cu and pc-Cu were measured in a gastight, H-cell separated by a proton exchange membrane with CO_2_-saturated 0.1 M KHCO_3_ (pH = 6.8) at room temperature and at atmospheric pressure. Tw-Cu and pc-Cu first were polished electrochemically in 85% H_3_PO_4_ solution and then were washed with DI water and immediately transferred to the H-cell prior to every CO_2_RR test. The CO_2_RR performance was analyzed at potentials ranging from −0.98 to −1.3 V vs RHE. The performances of tw-Cu and pc-Cu are summarized in Figure 2 and Tables S1 and S2. The dominant CO_2_RR products were gaseous CH_4_, ethylene (C_2_H_4_), carbon monoxide (CO), and hydrogen (H_2_). As the FE of liquid products was less than 1%, we focused our analysis on gas-phase products. Notably, tw-Cu showed initial production of CH_4_ from −0.99 V vs RHE, which reached the highest FE of 86.1% at −1.2 V vs RHE, doubling the observed FE of CH_4_ on pc-Cu (43.4%) at the same potential (−1.2 V vs RHE). Accordingly, tw-Cu showed larger absolute CH_4_ partial current densities (*j*_CH_4__) compared to pc-Cu. The *j*_CH_4__ of tw-Cu reached −21.7 mA/cm^2^ at −1.3 V vs RHE, a much larger magnitude than that of pc-Cu *j*_CH_4__ (−16.3 mA/cm^2^). Hereafter, comparisons between cathodic (partial) current densities, which by convention are negative, will refer to their magnitude only. The superior selectivity for CH_4_ on tw-Cu was accompanied by suppression of H_2_ and C_2_H_4_ generation. The H_2_ selectivity observed on tw-Cu is 10% less than that on pc-Cu from −1 to −1.2 V vs RHE. The partial current density of H_2_ (*j*_H_2__) on tw-Cu remained low at −1.8 mA/cm^2^ at −1.2 V vs RHE, while the *j*_H_2__ on pc-Cu significantly increased from −1.6 mA/cm^2^ (−1.0 V vs RHE) to −5.6 mA/cm^2^ (−1.2 V vs RHE), which suggests the lower intrinsic activity of the hydrogen evolution reaction (HER) on tw-Cu. Meanwhile, tw-Cu exhibited lower C_2_H_4_ selectivity than pc-Cu in the potential range of −1.1 to −1.3 V vs RHE. At −1.2 V vs RHE, the FE of C_2_H_4_ reached 39.6% on pc-Cu, which is 10 times higher than that of tw-Cu at the same potential. Similarly, the partial current density of C_2_H_4_ (*j*_C_2_H_4__) on pc-Cu reached −8.3 mA/cm^2^ at −1.2 V vs RHE, which is 9.22 times higher than that on tw-Cu (−0.9 mA/cm^2^). Taken together, tw-Cu showed a remarkably high FE_CH_4__ in an H-cell, not just when compared to pc-Cu but also when compared to single crystal Cu (111)^38^ and (110)^39^ surfaces, which indicates that the twin-boundary defect is critical in promoting the CH_4_ selectivity. Furthermore, the FE of CH_4_ on tw-Cu is superior to many state-of-the-art Cu-based catalysts reported in the literature, which includes fivefold twinned Cu nanowires (NWs),^40^ copper(II) phthalocyanine,^41^ nanotwinned copper,^42^ and Cu–Bi nanoalloys^43^ (Table 1).

**Table 1:** Comparison of CO_2_RR in Peak CH_4_ Production for Different Cu-Based Catalysts in H-Cells

catalyst	FE_CH_4__ (%)	applied potentials (V vs RHE)	electrolyte	reference
tw-Cu	86	–1.22	0.1 M KHCO_3_	this work
pc-Cu	43	–1.23	0.1 M KHCO_3_	
single crystal (111)	46	–1.15	0.1 M KHCO_3_	(38)
single crystal (110)	50	–1.15	0.1 M KHCO_3_	(39)
fivefold twinned Cu NWs loaded on carbon black	55	–1.25	0.1 M KHCO_3_	(40)
copper(II) phthalocyanine	66	–1.06	0.5 M KHCO_3_	(41)
nanotwinned copper	59^a^	–1.6	0.2 M KHCO_3_	(42)
Cu–Bi nanoalloys	70.6	–1.2	0.5 M KHCO_3_	(43)

a Ref (42) reported FE_CH_4__ = 92%; this value however is the so-called intrinsic FE that the authors estimated to be solely contributed by the twin boundaries (excluding the planar regions), i.e., not of the whole catalyst as is reported here. 59% is the reported peak FE_CH_4__ for the entire catalyst.

To study further the competition between the HER and the CO_2_RR on tw-Cu and pc-Cu, we performed linear sweep voltammetry (LSV) measurements in N_2_-saturated (not CO_2_RR, only HER) and CO_2_-saturated electrolyte, respectively (Figure S3). For tw-Cu, the total current density (−36.2 mA/cm^2^) increased in magnitude by 28.4 mA/cm^2^ at −1.2 V vs RHE in CO_2_-saturated electrolyte compared with N_2_-saturated electrolyte (Figure S3). On the other hand, the magnitude of the current density for pc-Cu increased by only 8.9 mA/cm^2^ at the same potential (Figure S3). The larger cathodic current density enhancement on tw-Cu in CO_2_-saturated electrolyte thus implies a higher CO_2_RR efficiency. Under N_2_-saturated conditions, however, pc-Cu exhibited a larger current density of −9.8 mA/cm^2^ at −1.2 V vs RHE compared to tw-Cu (−7.6 mA/cm^2^), indicating a higher HER activity on pc-Cu.

Interestingly, the high CH_4_ selectivity found on tw-Cu is distinct from CO_2_RR catalyzed on Cu rich in grain boundaries. Grain-boundary-rich Cu catalysts were reported to be moderately selective toward C_2_ products, with 30–45% FE toward C_2_H_4_OH, OAc-, or C_2_H_4_ without producing CH_4_ (Table S3).^19−22,44,45^ These different product distributions suggest that twin boundaries contain different active sites for CO_2_RR from those present at grain boundaries.

### Quantum Mechanical Studies of Activity and Selectivity

**Figure 3:**
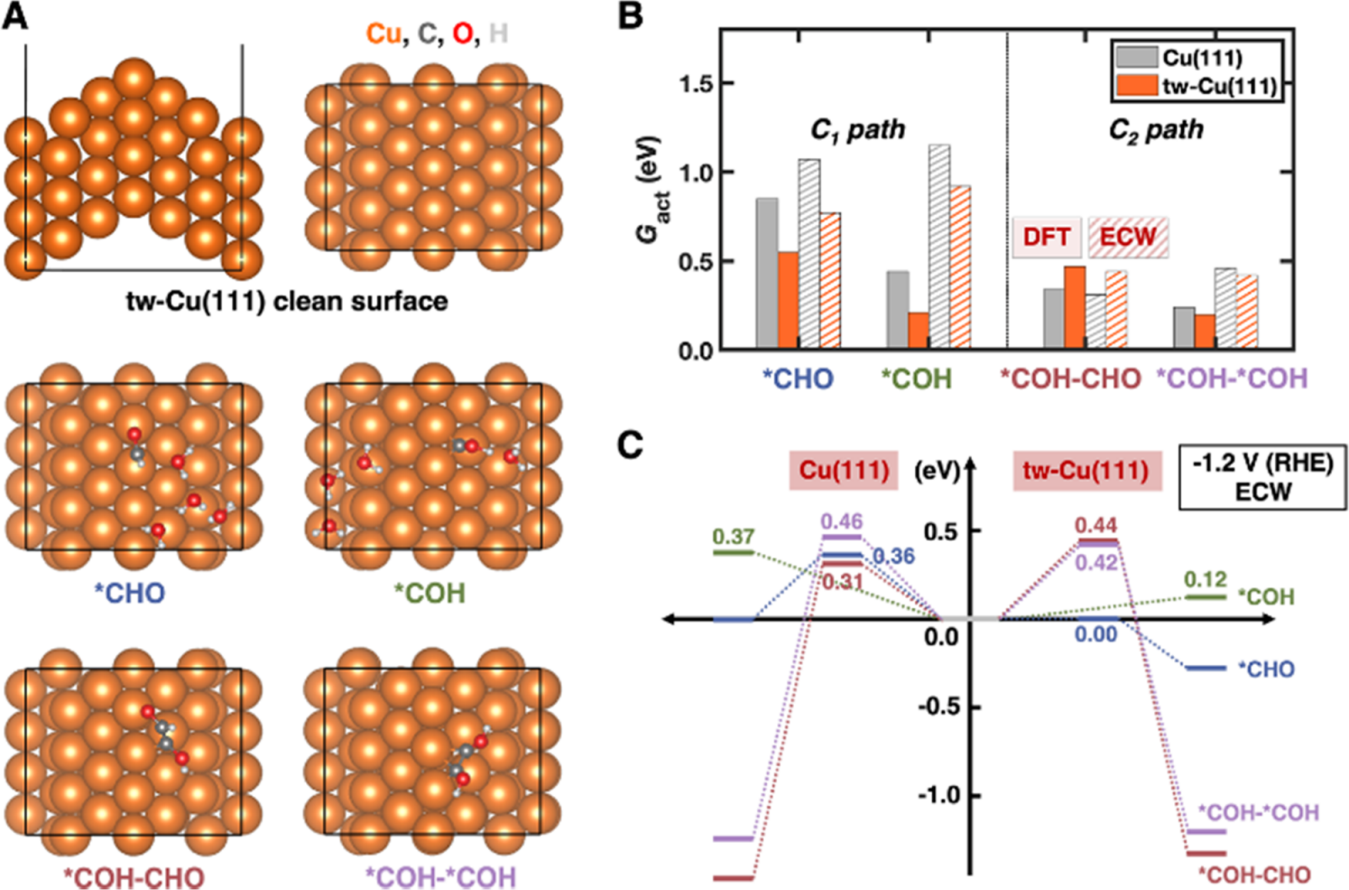
Quantum mechanical simulations of the rate-limiting steps of C_1_ and C_2_ pathways on tw-Cu(111). (A) (Top) Side (left) and top (right) views of the tw-Cu(111) surface periodic slab model and (middle and bottom panels) structures of products *CHO + (H_2_O)_4_, *COH + (H_2_O)_4_, *COH–CHO, and *COH–*COH (top views) as labeled. Cu in orange; C in dark gray; O in red; H in light gray. (B) Activation free energies *G*_act_ for C_1_ and C_2_ pathways on tw-Cu(111) (orange bars) and planar Cu(111) (gray bars) predicted by DFT-PBE-D3 (DFT; solid bars) and emb-CASPT2 (ECW; hatched bars) at constant-charge conditions. Both DFT-PBE-D3-derived and emb-CASPT2-derived barriers on planar Cu(111) were taken from refs (47, 48). The emb-CASPT2 results on tw-Cu(111) were estimated from energetic differences between emb-CASPT2 and DFT-PBE-D3 on planar Cu(111). Details are provided in Note S2. (C) Energetics of the *COH (green), *CHO (blue), *COH–*COH (purple), and *COH–CHO (red) pathways on planar Cu(111) (left) and tw-Cu(111) (right) predicted by emb-CASPT2 (ECW) at an applied potential of −1.2 V vs RHE (see Note S3).

To gain further insights into the highly improved selectivity toward CH_4_ production on tw-Cu, we first performed van-der-Waals-corrected periodic DFT calculations (DFT-PBE-D3, see the Experimental Methods section and Note S1) to determine the reaction barriers of pertinent reactions in CO_2_RR. Our previous high-level quantum mechanical simulations demonstrated that the rate-limiting step toward CH_4_ on Cu(111) likely involves reduction of adsorbed CO (*CO) roughly equally to hydroxymethylidyne (*COH) and formyl (*CHO) at −0.9 V vs RHE.^46,47^ In both hydrogenation reactions, a proton-coupled electron transfer (PCET) mechanism is preferred over surface hydride transfer.^46,47^ Furthermore, two adsorbed hydrogenated CO species (*COH and/or *CHO) are necessary reaction intermediates for C–C coupling toward multicarbon products on the same facet, and *COH–CHO and *COH–*COH are the most kinetically favorable coupling products.^48^ We therefore calculated the activation barriers for these same C_1_ and C_2+_ pathways on a Cu(111) slab with twin-boundary assemblies (tw-Cu(111)) (Figure 3A). The product structures and critical structures along the minimum-energy pathways (MEPs) appear, respectively, in Figures 3A and S4.

To simulate the rate-determining C_1_ path (i.e., CO hydrogenation via PCET) on tw-Cu(111), we introduced an Eigen cation (H_9_O_4_^+^) as a proton source to represent the explicit solvent. We predict at the DFT-PBE-D3 level that the reduction of *CO to form *CHO occurs with an activation (reaction) free energy of 0.55 (0.34) eV (Figure 3B). The competing *CO reduction to form *COH is a transition-state-free process with an activation and reaction free energy at the DFT-PBE-D3 level of 0.21 eV (Figure 3B). Compared with the DFT-PBE-D3 activation barriers of 0.85 eV for *CHO formation and 0.44 eV for *COH formation on planar Cu(111),^47^ tw-Cu(111) exhibits lower activation barriers for both *CO reduction steps. Given that the first hydrogenation step is likely rate-determining in CO_2_RR, this may explain the observed higher CO_2_RR reaction rate and the enhanced CH_4_ production on tw-Cu.

We next calculated the barriers for C–C coupling to understand the selectivity toward C_1_ (CH_4_) vs C_2_ (C_2_H_4_) products on tw-Cu(111). C–C coupling routes are nonelectroactive, and thus no H_9_O_4_^+^ was included in the simulations. The predicted barrier at the DFT-PBE-D3 level for forming *COH–CHO is 0.47 eV, which is higher than the 0.34 eV on planar Cu(111)^48^ (Figure 3B). The DFT-PBE-D3 barrier for the other C–C coupling step forming *COH–*COH decreases only slightly to 0.20 eV on tw-Cu(111) compared to 0.24 eV on planar Cu(111)^48^ (Figure 3B). Because tw-Cu(111) maintains similar or higher barriers for C–C coupling (a C_2_ rate-limiting step), whereas above we show that hydrogenation of *CO is promoted significantly by the twin boundaries, the C_1_ path toward CH_4_ may be disproportionately enhanced. However, because PCET is involved, rigorously analyzing the selectivity toward CH_4_ vs C_2_H_4_ requires a potential-dependent barrier analysis under working conditions (vide infra).

We showed previously that one needs to use embedded correlated wavefunction (ECW) theory^49−53^ to predict accurately the activity and selectivity of CO_2_RR on planar Cu(111).^46,47^ We therefore expect that the same level of theory is needed to describe correctly CO_2_RR on tw-Cu(111). However, the high computational cost of, e.g., emb-CASPT2^54,55^ impedes such investigations. We therefore used the energetic differences predicted between emb-CASPT2 and DFT-PBE-D3 on planar Cu(111)—an ECW correction—to approximate emb-CASPT2-predicted barriers on tw-Cu(111) (Note S2 and Table S4). In other words, we shifted the reaction and activation energies on tw-Cu(111) by the same ECW correction as their counterpart reaction on planar Cu(111). The nature of the difference in the predicted activation barriers between emb-CASPT2 and DFT-PBE-D3 in part originates from the difference in their descriptions of charge transfer during a reaction. We reached this conclusion by establishing a good linear correlation between (activation and reaction) free energy differences and charge change differences on adsorbates predicted at the two levels of theory on planar Cu(111) (Note S2, Tables S5 and S6, and Figure S5). Because the same pathway on two different surfaces involves the same amount of charge transferred from the surface to the adsorbates and vice versa, we may directly use the corrections obtained from planar Cu(111) on tw-Cu(111)—foregoing the need to perform expensive emb-CASPT2 calculations on tw-Cu(111) (Table S7). By applying this strategy, emb-CASPT2 would predict that the reduction of *CO to *CHO (*COH) via PCET occurs with a barrier of 0.77 (0.92) eV on tw-Cu(111), lower than the barrier of 1.07 (1.15) eV on planar Cu(111)^47^ (Figure 3B). In contrast, emb-CASPT2 would predict that the preferred C–C coupling route is the formation of *COH–*COH on tw-Cu(111) with a barrier of 0.42 eV, higher than the activation barrier of 0.31 eV for the most preferred *COH–CHO pathway on planar Cu(111)^48^ (Figure 3B). Therefore, using this beyond DFT ECW theory that properly treats charge transfer, we again predict that the use of tw-Cu(111) rather than planar Cu(111) reduces barriers for C_1_ rate-limiting steps, while increasing barriers for C–C coupling to form the most favored product (*COH–*COH on tw-Cu(111) vs *COH–CHO on planar Cu(111)).

Finally, to fully rationalize selectivity toward CH_4_ vs C_2_H_4_ on tw-Cu(111), we contextualized the activation free energies presented above under real electrochemical conditions by transforming them from being a function of charge to a function of electrochemical potential (a thermodynamic Legendre transformation from constant charge to constant electrochemical potential), to determine potential dependence of the barriers (Note S3, Figure S6, and Tables S8 and S9).^47^ We performed this analysis for electroactive CO PCET reduction steps. The emb-CASPT2 activation barriers at an applied potential of −1.2 V vs RHE (Figure 3C and Table S9) for C_1_ rate-determining steps (0.00 eV for *CHO formation and 0.12 eV for *COH formation) are lower than that of the C_2_ rate-determining steps (0.42 eV for *COH–*COH formation and 0.44 eV for *COH–CHO formation) on tw-Cu(111). These trends illustrate that tw-Cu(111) can enhance CH_4_ production via substantially accelerated CO hydrogenation kinetics. By contrast, barriers for C–C coupling do not decrease on tw-Cu(111), effectively limiting C_2_H_4_ formation at this applied potential and explaining the high (low) FE for CH_4_ (C_2_H_4_) observed in the experiment (Figure 2). Unlike tw-Cu(111), planar Cu(111) exhibits similar emb-CASPT2 barriers for C_1_ (0.36 eV for *CHO formation and 0.37 eV for *COH formation) and C_2_ (0.46 eV for *COH–*COH formation and 0.31 eV for *COH–CHO formation) rate-determining steps at the same applied potential (Figure 3C). This would explain the almost identical FEs, and thus a similar degree of preference for CH_4_ and C_2_H_4_, observed at −1.2 V vs RHE for pc-Cu (Figure 2).

## Conclusions

To summarize, we report that a tw-Cu catalyst with a densely packed TBs on the surface exhibits a high FE_CH_4__ (86.1 ± 5.3%) in an H-cell. Coupled with structural and electrochemical surface characterizations of the tw-Cu catalyst, our computational analysis showed that the existence of TBs in Cu(111) electrodes decreases the barriers for CO hydrogenation, while not doing so for C–C coupling, leading to a higher selectivity toward CH_4_ over C_2_ products. Our findings suggested an effective approach for tuning CO_2_RR product selectivity by catalyst surface structure engineering.

## Supporting Information Available

Computational details, characterization data, and catalytic performance data, including the schematic diagram for the synthesis of the tw-Cu; SEM image of tw-Cu and pc-Cu; linear sweep voltammetry curves obtained on tw-Cu and pc-Cu in N_2_-saturated and CO_2_-saturated electrolytes; simulation structures; plot of the predicted free energy difference vs the adsorbate electron charge change difference between emb-CASSCF and emb-DFT-PBE cluster models; potential dependence of the free energy for CO reduction to form *CHO and *COH via PCET on tw-Cu(111); DFT-PBE-D3 identified adsorption structures and sites of *CO, *CHO, *COH, *COH–CHO, and *COH–*COH on tw-Cu(111); and summary of FE at all applied potentials on tw-Cu and pc-Cu (PDF)
