# Supplementary material for: Highly Selective Electrochemical Reduction of CO2 into Methane on Nanotwinned Cu
Source: J Am Chem Soc. 2023 Apr 18;145(16):9136–43. doi: 10.1021/jacs.3c00847 (PMC10141442; doi:10.1021/jacs.3c00847)
Supplement: Supplementary file 1 — ja3c00847_si_001.pdf [file ja3c00847_si_001.pdf]

Supporting Information

Highly Selective Electrochemical Reduction of CO_2 into Methane on Nanotwinned Cu

Jin Cai,[1] Qing Zhao,[2,3] Wei-You Hsu,[4] Chungseok Choi,[1] Yang Liu,[1] John Mark P. Martirez,[5,7] Chih Chen,[4] Jin Huang,[1] Emily A. Carter,[2,5*] and Yu Huang[1,6*]

[1] Department of Materials Science and Engineering, University of California, Los Angeles, Los Angeles, CA, 90095-1595, United States

[2] Department of Mechanical and Aerospace Engineering and the Andlinger Center for Energy and the Environment, Princeton University, Princeton, NJ, 08544-5263, United States

[3] Present address: Department of Chemical Engineering, Northeastern University, Boston, MA, 02115-5005, United States

[4] Department of Materials Science and Engineering, National Yang Ming Chiao Tung University, Hsinchu, Taiwan 30010, ROC

[5] Department of Chemical and Biomolecular Engineering, University of California, Los Angeles, Los Angeles, CA, 90095-1592, United States

[6] California NanoSystems Institute, University of California, Los Angeles, Los Angeles, CA, 90095, United States

[7] Present address: Applied Materials and Sustainability Sciences, Princeton Plasma Physics Laboratory, Princeton, New Jersey 08540, United States

*Corresponding author. Email: yhuang@seas.ucla.edu (Y.H.), eac@princeton.edu (E.A.C)

This PDF file includes:

Supplementary Notes
Figs. S1 to S7
Tables S1 to S10
References (1 to 21)

Supplementary Notes

Note S1. Computational details

In the CI-NEB calculations, we applied an artificial spring force constant of 3 eV $Å^{-2}$ along the elastic band tangents. We started from the preferred adsorption sites for both reactants and products. Details of screening adsorption sites for key intermediates are discussed below. We included an Eigen cation ($H_9O_4^+$) as explicit solvent and proton source in optimizing the reaction MEPs for *CO PCET reduction to form *CHO and *COH. In addition to the explicit water molecules, we also considered long-range solvation effects by performing single-point energy calculations with an implicit continuum solvation model[1-2] applied in the vacuum region. Here, we employed a polarizable dielectric continuum model[1-2] implemented in VASP with a dielectric constant of 78.4 to represent the pure liquid water. We showed previously that structurally optimizing CO hydrogenation pathways on planar Cu(111) in the presence of an implicit continuum solvation model leads to similar structures and energies relative to optimization in vacuum[3], and thus here we only included the long-range solvation effects in subsequent single-point total energy calculations. We did not apply dipole-field energy and potential corrections when using the implicit solvation model because this model itself screens the surface dipole from its dielectric response. For non-electroactive C-C coupling steps (*COH-CHO and *COH-*COH pathways), we did not include any explicit water molecule and did not apply the implicit solvation model in the MEPs. The methods of treating solvent are consistent with our previous study on planar Cu(111).[3-4]

We calculated the activation and reaction free energies by including zero-point energies, thermal corrections, and entropy terms (at 298.15 K) using the harmonic oscillator approximation. We included the vibrational contributions of all adsorbates, water and hydronium (H_3O^+) molecules, and Cu atoms directly bonded to the adsorbates. We computed vibrational frequencies by constructing Hessian matrices numerically from a first-order finite-difference scheme using the structures optimized in vacuum (i.e., without implicit continuum solvent). In principle, the optimized initial (reactant) and final (product) state structures are local minima and thus should not exhibit imaginary vibrational modes, whereas the optimized transition-state structure should exhibit a single imaginary vibrational mode. However, we observed several small imaginary frequencies below 80*i* cm^{-1} for some of the critical structures in addition to the transition-state's primary imaginary mode in the two CO reduction reactions: 23*i* cm^{-1}, 33*i* cm^{-1}, 48*i* cm^{-1}, and 59*i* cm^{-1} for the initial-state structure of the CO reduction to form *COH and *CHO; 8*i* cm^{-1}, 40*i* cm^{-1}, 51*i* cm^{-1}, and 75*i* cm^{-1} for the transition-state structure along the MEP of the CO reduction to form *CHO; 17*i* cm^{-1} and 46*i* cm^{-1} for the final-state structure of the CO reduction to form *CHO ; 25*i* cm^{-1}, 39*i* cm^{-1}, 40*i* cm^{-1}, and 52*i* cm^{-1} for the final-state structure of the CO reduction to form *COH. These small imaginary frequency modes correspond to rotations of the explicit solvent

molecules ($H_9O_4^+$) included in the MEP calculations and are not involved in the proton-transfer mechanisms. Previous work[5-6] demonstrated that further structure refinements cannot remove such small imaginary modes corresponding to solvent rotations and that the influence on the energetics are negligible. Therefore, we deemed all those small imaginary modes reported above to be numerical noise and ignored them in our free-energy calculations.

We screened possible adsorption sites of *CO, *COH, *CHO, *COH-CHO, and *COH-*COH on tw-Cu(111) using DFT-PBE-D3. We examined the two possible *CO adsorption sites on the twin boundary region between two Cu(111) surfaces: atop and bridge. In addition to that, we also examined the other two possible *CO adsorption sites close to the boundary region, i.e., on one of the Cu(111) surfaces: hexagonal-close-packed (hcp) and face-centered-cubic (fcc) threefold-hollow sites. DFT-PBE-D3 exhibits a preference for the bridge site on the twin boundary region. We then compared the same four possible *COH adsorption sites on tw-Cu(111); DFT-PBE-D3 predicts that the fcc-hollow site is the most stable. We next investigated the adsorption of *CHO. In addition to the four possible adsorption sites studied above for *COH and *CO, *CHO can also reside on top of two adjacent Cu atoms on the twin boundary region with the C–O bond nearly parallel to the twin boundary Cu–Cu bond, forming a dative bond from an O lone pair to one of the Cu atoms, denoted as a bidentate atop site. DFT-PBE-D3 predicts that the bidentate atop site is preferred for *CHO. For *COH-CHO, we considered the DFT-PBE-D3-preferred bridge site on planar Cu(111) established in previous work[4] and an additional atop site on the twin boundary region. DFT-PBE-D3 predicts that the bridge site close to the twin boundary region on one of the Cu(111) surfaces is more favorable for the tw-Cu(111). For *COH-*COH, we considered again the DFT-PBE-D3-preferred fcc-hcp site on planar Cu(111) observed in our previous work[4] and the new atop-atop site on the boundary region. DFT-PBE-D3 predicts that the fcc-hcp site close to the twin boundary region on one of the Cu(111) surfaces is preferred for the tw-Cu(111). Structures and relative energies for all of the examined adsorption sites of each adsorbate on tw-Cu(111) are shown in Figure S7 and Table S10, respectively. The configurations of the DFT-PBE-D3-predicted adsorption sites of each adsorbate are shown as either initial-state structures or final-state structures in Figure S4.

Note S2. Approximate emb-CASPT2 barriers on tw-Cu(111).

Standard exchange-correlation functionals, such as the widely used PBE functional, used in DFT suffer from electron self-interaction error and a lack of energy derivative discontinuity.[7] The self-interaction error induces over-delocalization of electron density and thus generally fails to describe charge-transfer processes adequately. The quantitative inaccuracy of DFT-PBE-D3 energetics thus originates at least in part from its inadequate description of charge-transfer processes, i.e., the charge redistribution as the reactant transforms into the transition state or product. This in turn adversely affects the accuracy of activation and reaction free energies. For the four reactions (CO reduction to form *CHO and *COH, and *COH-CHO and *COH-*COH C-C coupling) evaluated by DFT-PBE-D3 on tw-Cu(111) in this work, we had computed previously their activation and reaction free energies using both DFT-PBE-D3 and emb-CASPT2 methods on planar Cu(111).[3-4] These prior calculations enabled us to verify this charge-transfer-error-to-energy-error correlation. The energetics of the aforementioned four reactions evaluated by both DFT-PBE-D3 and emb-CASPT2 on planar Cu(111)[3-4] are tabulated in Table S5. To represent the charge evolution along a reaction, we calculated differences in predicted overall charge changes on the adsorbates, using Bader charge analysis,[8-9] between the embedded complete active space self-consistent field[10-11] (emb-CASSCF) and embedded DFT-PBE (emb-DFT-PBE) electron densities. Bader charge changes and the deviation between the two methods for the abovementioned four reactions on planar Cu(111) were taken from our previous studies,[3-4] which are summarized in Table S6. We used the electron densities from the emb-DFT-PBE cluster model instead of from the DFT-PBE-D3 slab model, to approximate changes in the partitioned electron charge on the adsorbates at the DFT-PBE level for consistency in the basis set when comparing CASSCF and DFT-PBE charges.

We observed a linear correlation ($R^2 = 0.78$) between energy differences predicted by emb-CASPT2 vs. DFT-PBE-D3 and differences in the predicted change in electron charge on the adsorbates calculated using emb-CASSCF vs. emb-DFT-PBE cluster electron densities for activation and reaction free energies (Figure S5). These results verify our hypothesis that the quantitative inaccuracy of DFT-PBE-D3 energetics largely originates from its inadequate description of charge transfer. We then use the energetic differences between emb-CASPT2 and DFT-PBE-D3 on planar Cu(111) to approximate the emb-CASPT2-derived energetics on tw-Cu(111), where we assume the same charge-transfer error persists on the tw-Cu(111) surface for the same type of reaction (Table S4). In Table S7 we show that the same reaction occurring on planar Cu(111) and tw-Cu(111) results in similar charge-transfer change at its transition state and product within DFT-PBE-D3. This provides additional credence to our assumption that similar charge-transfer error will be incurred on tw-Cu(111) as in planar Cu(111), therefore warranting similar energy error corrections.

Note S3. Potential-dependent activation and reaction free energies.

We describe the method to simulate potential-dependent activation and reaction free energies in our recent work[3] about predicting CO_2RR mechanisms on planar Cu(111). For completeness, we outline the details below in the context of the mechanisms on tw-Cu(111) evaluated in this work.

We determine potential-dependent reaction and activation free energies by combining Chan and Nørskov's capacitor model[12-13] and the computational hydrogen electrode (CHE) model[14] developed by Nørskov *et al.* We first convert the simulated constant-charge conditions to the experimental electrochemical constant-potential conditions using the Chan-Nørskov capacitor model[12-13]. However, here, we use the surface potential relative to the bulk continuum solvent's energy level ($\Delta\Phi$), instead of referencing to the vacuum level (i.e., the work function, Φ) as in the original formulation. This is because we calculated the free energies in the presence of an implicit continuum solvation model of water. Li *et al.*[15] demonstrated the two formulations to be equivalent. The free energy changes between any two states (i.e., state 1 and state 2) at constant relative work functions are given by

$$G_2(\Delta\Phi_1) - G_1(\Delta\Phi_1) = G_2(\Delta\Phi_2) - G_1(\Delta\Phi_1) - \frac{(q_2 - q_1)(\Delta\Phi_2 - \Delta\Phi_1)}{2}$$

$$G_2(\Delta\Phi_2) - G_1(\Delta\Phi_2) = G_2(\Delta\Phi_2) - G_1(\Delta\Phi_1) + \frac{(q_2 - q_1)(\Delta\Phi_2 - \Delta\Phi_1)}{2}$$

in which $G_1(\Delta\Phi_1)$ and $G_2(\Delta\Phi_2)$ are the simulated DFT-PBE-D3 free energies at constant charge conditions, $\Delta\Phi_1$ and $\Delta\Phi_2$ are the work functions relative to the bulk (water) solvent energy level, and q_1 and q_2 are the surface charges, at states 1 and 2. q is the charge of the tw-Cu(111) slab including all adsorbates calculated using a Bader analysis.[8-9] For activation (reaction) free energies, states 1 and 2 correspond to reactant and transition (product) states, respectively. Table S8 summarizes the computed relative work functions, surface Bader charges, and free energies at constant $\Delta\Phi$ at the DFT-PBE-D3 and emb-CASPT2 levels of theory for CO reduction to form *CHO and *COH via PCET on tw-Cu(111).

After establishing the linear dependence of the free energies on the relative work functions, we then determined the free energies referenced against the reversible hydrogen electrode (RHE). To achieve this, we calculate the DFT-PBE-D3 reaction free energies using the CHE model at an applied potential of 0.0 V vs. RHE using the equation:

$$\Delta G_{\mathrm{CHE}} = G(\mathrm{P}) - G(\mathrm{R}) - \tfrac{1}{2} G(\mathrm{H_2}),$$

in which G(P), G(R), and $G(H_2)$ are free energies of the product, reactant, and a gas-phase H_2 molecule simulated in vacuum, respectively. The product is the tw-Cu(111) slab with a *CHO (*COH) adsorbate and four explicit water molecules subject to an implicit continuum solvent for *CO reduction, while the reactant is the tw-Cu(111) slab with a *CO adsorbate and four explicit

water molecules subject to an implicit continuum solvent. The calculated reaction free energy using the CHE model for CO reduction to form *CHO (*COH) path is 0.54 (0.41) eV.

We then locate the $\Delta\Phi$ associated with the DFT-PBE-D3 reaction free energy computed using CHE model at 0.0 V vs. RHE by $\Delta G(\Delta\Phi) = \Delta G_{\mathrm{CHE}}$ at $\Delta\Phi(U = 0\ \mathrm{V})$. U is the electrode potential relative to the RHE. In other words, $\Delta\Phi(U = 0\ \mathrm{V})$ is the value of $\Delta\Phi$ at which the horizontal line $\Delta G = \Delta G_{\mathrm{CHE}}$ and DFT-PBE-D3 line $\Delta G = \Delta G(\Delta\Phi)$ intersect. Then we can locate any applied potential vs. RHE using the corresponding relative work function $\Delta\Phi(U) = \Delta\Phi(U = 0\ \mathrm{V}) + eU$. The corresponding value of the reaction (activation) free energy at $\Delta\Phi(U)$ defines the reaction (activation) free energy at U; the reaction (activation) free energy at U is found as the intersection of the vertical line defining $\Delta\Phi(U)$ and the line $\Delta G = \Delta G(\Delta\Phi)$ ($G_{\mathrm{act}} = G_{\mathrm{act}}\ (\Delta\Phi)$).

Figure S6A shows the detailed steps to simulate the free energies of *CO reduction to form *CHO at an applied potential of -1.2 V vs. RHE. We first used the two points (filled circles for reaction free energies, empty circles for activation free energies) at constant $\Delta\Phi$ (derived from $\Delta\Phi_R$ and $\Delta\Phi_P$ for reaction free energies, $\Delta\Phi_R$ and $\Delta\Phi_{TS}$ for activation free energies, as discussed above), marked by the vertical dotted black lines in Figure S6A, to establish the linear dependence of the free energies on $\Delta\Phi$ for the tw-Cu(111) surface. The linear fits allow us to compute the reaction (activation) free energy at any $\Delta\Phi$. To determine the free energies at applied potentials referenced against the RHE instead of the relative work function, $\Delta\Phi$, we employ the DFT-PBE-D3 reaction free energies computed using the CHE model at an applied potential of 0.0 V vs. RHE (0.54 eV for the *CHO path and 0.41 eV for the *COH path). We obtained the intersection (red triangles in Figure S6A) between the reaction free energy of 0.54 eV from CHE model for *CHO formation (horizontal dashed black line in Figure S6A) and the red solid line that represents the $\Delta\Phi$-dependent DFT-PBE-D3 reaction free energy, and then assigned the intersection point as the $\Delta\Phi$ associated with an applied potential of 0.0 V vs. RHE (vertical dashed black line marked as 0.0 V vs. RHE in Figure S6A). We then shift the $\Delta\Phi$ by -1.2 eV to locate the applied potential of -1.2 V vs. RHE (vertical dashed black line marked as -1.2 V vs. RHE in Figure S6A). The intersection between this vertical line and the red dotted line representing the $\Delta\Phi$-dependent DFT-PBE-D3 activation free energy corresponds to the DFT-PBE-D3 activation free energy (red empty square in Figure S6A) at an applied potential of -1.2 V vs. RHE. Similarly, we obtained the emb-CASPT2 reaction (activation) free energy at an applied potential of -1.2 V vs. RHE by locating the intersection between this vertical line and the blue solid (dotted) line representing the $\Delta\Phi$-dependent emb-CASPT2 reaction (activation) free energy. We followed the same procedure to simulate the reaction free energy at -1.2 V vs. RHE for CO reduction via PCET to form *COH.

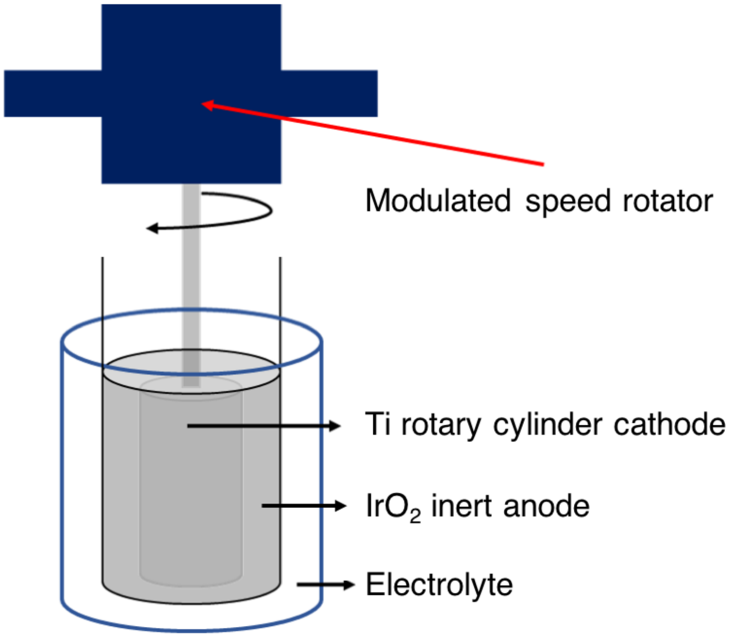

Fig. S1. Schematic diagram for the synthesis of the tw-Cu rotary electroplating system. The anode was titanium (Ti) coated with iridium dioxide (IrO_2) and the cylinder cathode was made of Ti. During the electroplating process, the cathode rotation speed was 800 rpm controlled by a modulated speed rotator. Tw-Cu was electroplated under a current density of 11 A dm^{-2} (ASD).

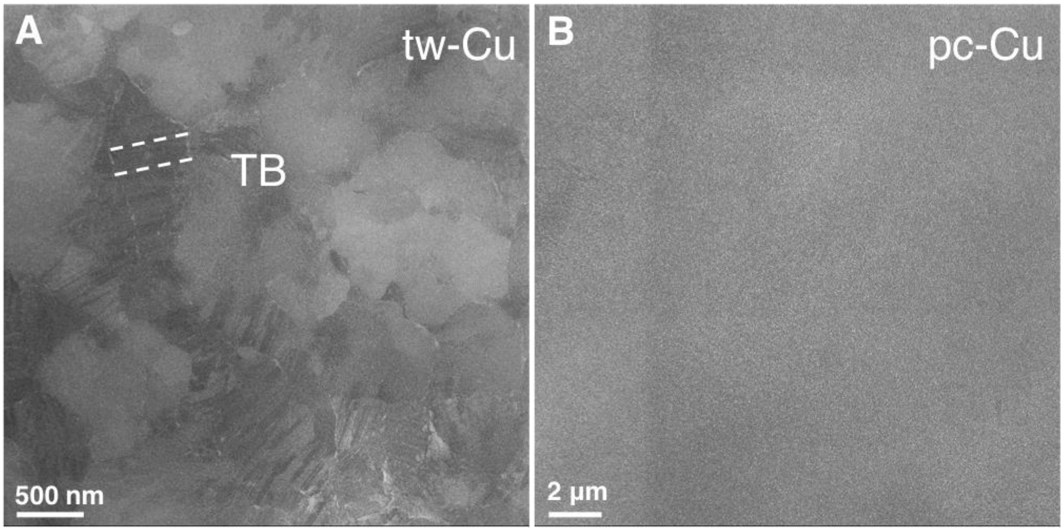

Fig. S2. SEM image of (A) tw-Cu. The white dashed lines mark the twin-boundary assembly. (B) Pc-Cu exhibits a relatively feature-less surface.

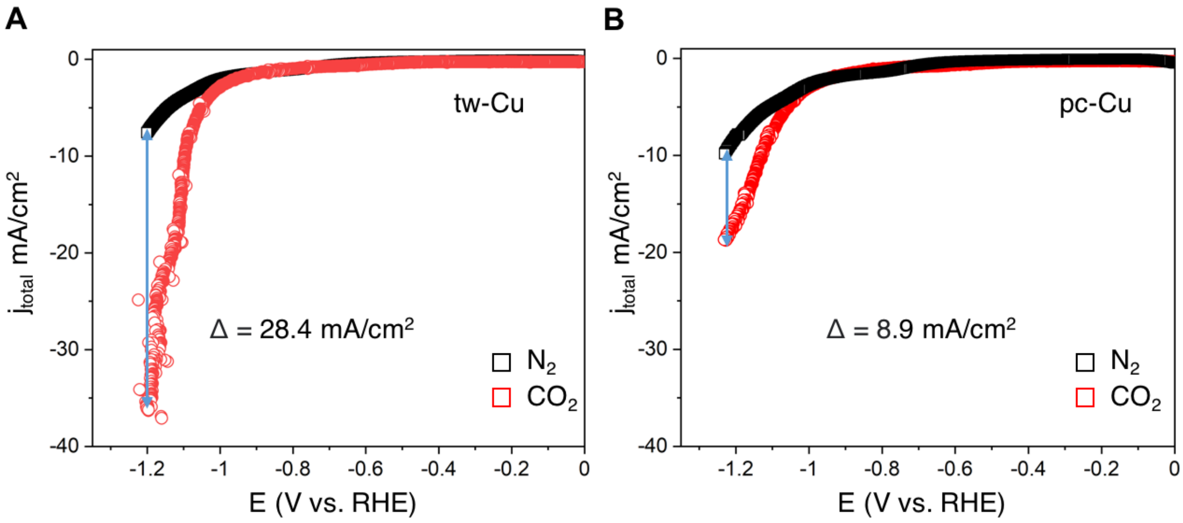

Fig. S3. Linear sweep voltammetry curves obtained on (A) tw-Cu and (B) pc-Cu in N_2-saturated and CO_2-saturated electrolytes. For tw-Cu, there is a clear current density enhancement in the CO_2-saturated solution in comparison with the N_2-saturated solution. The current density difference at −1.2 V vs. RHE is 28.4 mA cm^{-2}. For pc-Cu, the current density difference at −1.2 V vs. RHE is about 8.9 mA cm^{-2}. The larger current density difference suggests a more efficient CO_2RR on tw-Cu.

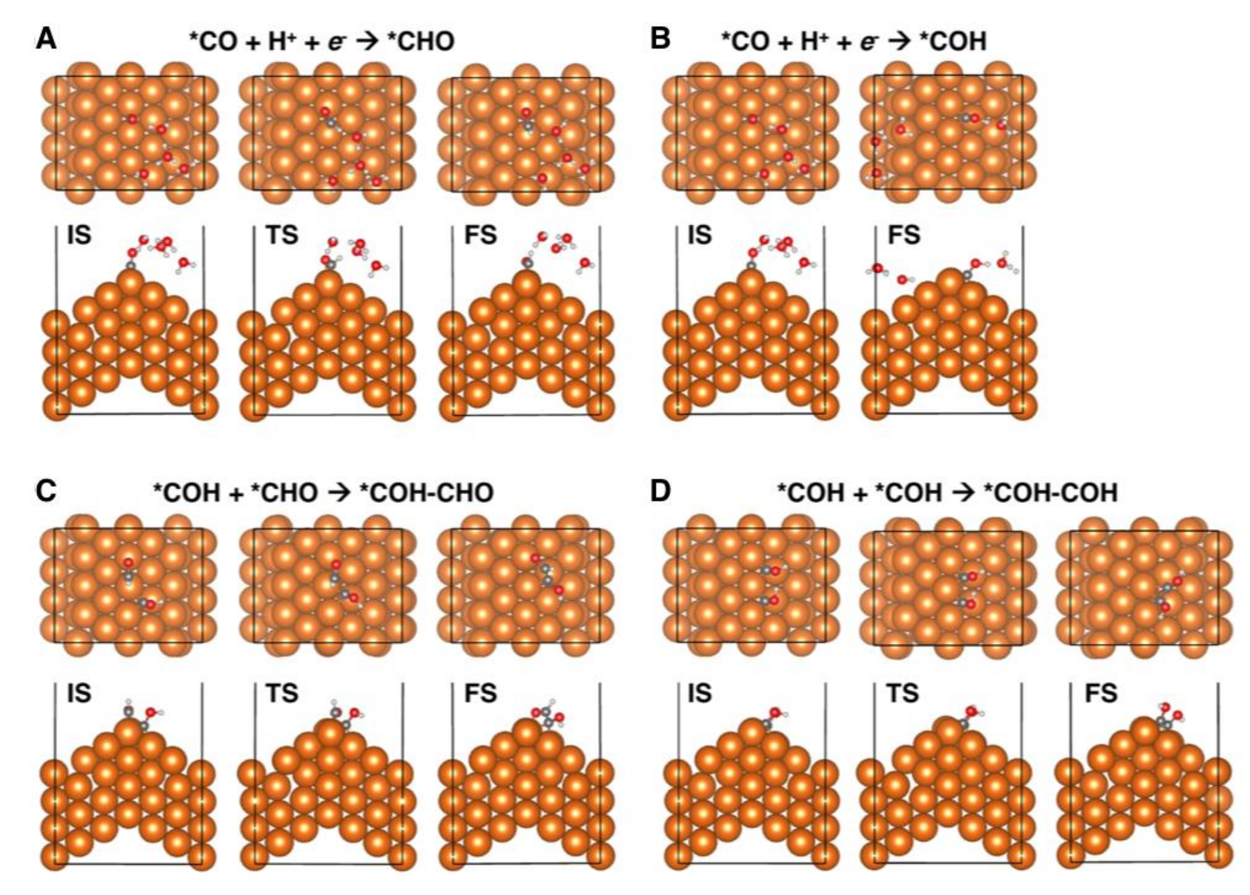

Fig. S4. Critical (initial-state (IS), transition-state (TS), and final-state (FS)) structures (top and side views in upper and lower panels, respectively) along the minimum energy paths optimized within DFT-PBE-D3 for: CO reduction via PCET to form (A) *CHO and (B) *COH, and (C) *COH-CHO and (D) *COH-*COH C-C coupling steps on tw-Cu(111). Atoms are colored as follows: Cu in orange, C in dark gray, O in red, and H in light gray.

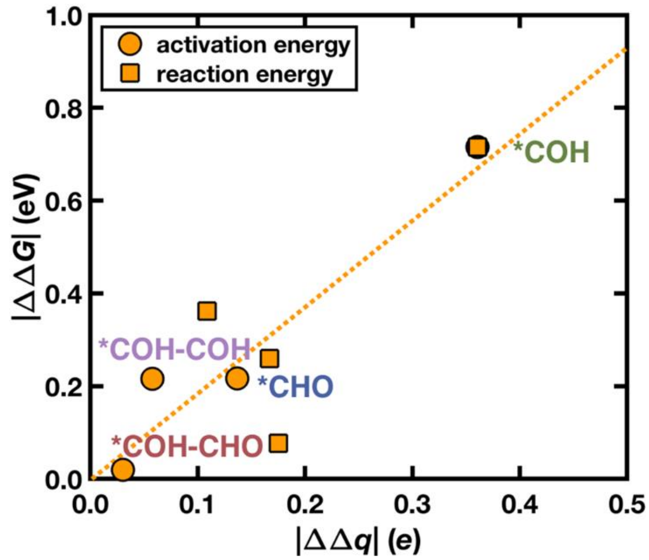

Fig. S5. Plot of the absolute value of the predicted free energy difference of reaction (squares) and activation (circles) between emb-CASPT2 and DFT-PBE-D3 ($|\Delta\Delta G|$) vs. the absolute value of adsorbate electron charge change difference between emb-CASSCF and emb-DFT-PBE cluster models ($|\Delta\Delta q|$) for CO reduction to form *CHO and *COH via PCET and C-C coupling steps to form *COH-CHO and *COH-*COH on planar Cu(111). We calculated the $|\Delta\Delta q|$ using Bader charge changes of adsorbates between transition (product) and reactant states. Only the results corresponding to the activation free energies (circles) are labelled. The energetics and structures to compute Bader charges for CO reduction to form *CHO and *COH, and those for *COH-CHO and *COH-*COH C-C coupling pathways were taken from Ref. 3 and Ref. 4, respectively.

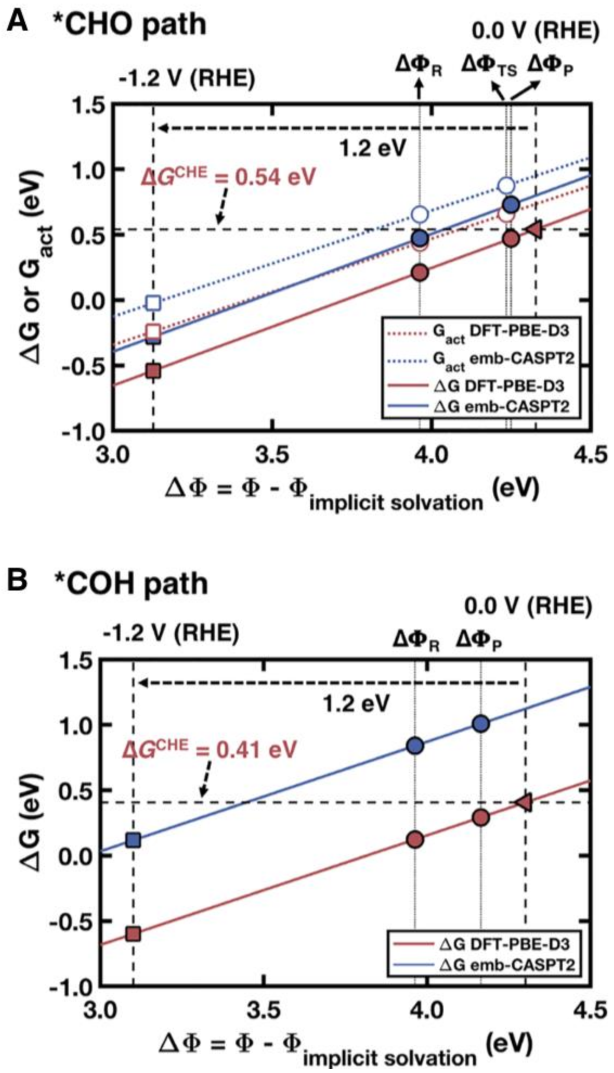

Fig. S6. Potential dependence of the reaction (ΔG) and activation (G_{act}) free energy for CO reduction to form (A) *CHO and (B) *COH via PCET on tw-Cu(111), as predicted by DFT-PBE-D3 (red lines) and emb-CASPT2 (blue lines). The DFT-PBE-D3 energies were calculated by applying *a posteriori* implicit continuum solvation corrections to the energies of vacuum-optimized structures. The emb-CASPT2 energies were approximated by applying corrections of the energy differences between emb-CASPT2 and DFT-PBE-D3 results on planar Cu(111) to the DFT-PBE-D3 energies. Circles represent reaction (filled symbols) and activation (empty symbols) free energies extracted from the Chan-Nørskov capacitor model[12-13] using DFT-PBE-D3 (red symbols) or emb-CASPT2 (blue symbols) free energies, DFT-PBE-D3 relative work functions ($\Delta\Phi$), and surface Bader charges as input (see Table S8). The horizontal black dashed lines indicate reaction free energies computed using a CHE model and DFT-PBE-D3 at an applied potential of 0.0 V vs. RHE. Triangles represent the intersections of these horizontal lines with their corresponding $\Delta\Phi$-dependent DFT-PBE-D3 reaction free energy lines. The vertical black dashed lines indicate $\Delta\Phi$s associated with applied potentials of 0.0 and -1.2 V vs. RHE as labelled. Squares represent data extrapolated to an applied potential of -1.2 V vs. RHE.

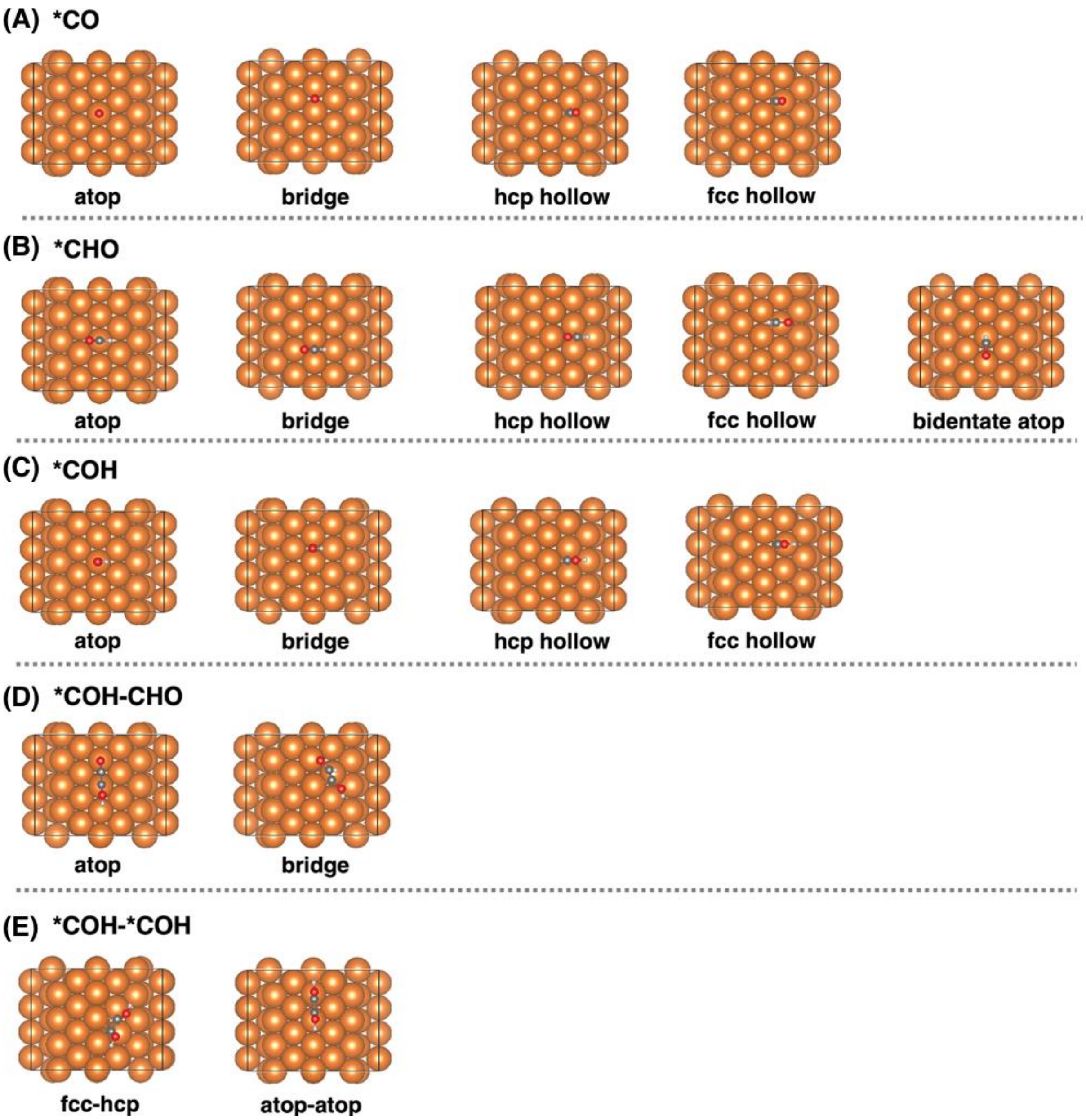

Fig. S7. DFT-PBE-D3 identified stable or metastable adsorption structures and sites of (a) *CO, (b) *CHO, (c) *COH, (d)*COH-CHO, and (e) *COH-*COH on tw-Cu(111). Atoms are colored as follows: Cu in orange, C in dark gray, O in red, and H in light gray.

Table S1. A summary of Faradaic efficiency (FE) at all applied potentials (E) vs. RHE on tw-Cu. The total FE is calculated by sum of FE of all products at each applied potential vs. RHE. Error bars correspond to the standard deviation of three independent measurements.

E (V vs. RHE)	Current Density mA/cm^2	FE$_{H_2}$ (%)	FE$_{CO}$ (%)	FE$_{CH_4}$ (%)	FE$_{C_2H_4}$ (%)	FE$_{HCOO^-}$ (%)	FE$_{CH_3CH_2\text{-}OH}$ (%)	Total FE (%)
-0.99 ±0.03	-2.30 ±1.61	49.16 ±9.35	32.12 ±14.91	4.67 ±8.09	8.08 ±13.99	0.04	0	94.07 ±2.49
-1.09 ±0.02	-5.66 ±0.51	24.72 ±6.33	19.24 ±5.40	27.25 ±3.93	22.91 ±6.43	0.09	0.09	94.30 ±6.44
-1.16 ±0.00	-11.87 ±1.36	11.77 ±2.52	8.07 ±4.80	61.59 ±4.00	18.75 ±3.12	0.09	0.13	100.41 ±7.36
-1.22 ±0.02	-20.53 ±2.20	8.75 ±6.93	2.34 ±2.22	86.08 ±5.34	4.43 ±0.96	0.01	0.05	101.65 ±2.36
-1.30 ±0.03	-31.39 ±2.55	39.36 ±10.24	1.19 ±1.14	69.51 ±4.94	0.74 ±1.28	0.01	0.04	110.86 ±5.81

Table S2. A summary of Faradaic efficiency (FE) at all applied potentials (E) vs. RHE on pc-Cu. The total FE is calculated by sum of FE of all products at each applied potential vs. RHE. Error bars correspond to the standard deviation of three independent measurements.

E (V vs. RHE)	Current Density mA/cm^2	FE_{H_2} (%)	FE_{CO} (%)	FE_{CH_4} (%)	$FE_{C_2H_4}$ (%)	FE_{HCOO^-} (%)	$FE_{CH_3CH_2\text{-}OH}$ (%)	Total FE (%)
-0.98 ±0.03	-2.70 ±0.63	59.81 ±10.96	27.73 ±9.21	0.00 ±0.00	5.70 ±4.98	0.35	0	93.24 ±1.27
-1.10 ±0.03	-7.95 ±2.71	34.46 ±10.22	9.43 ±3.48	22.36 ±0.37	30.29 ±6.53	0.04	0	96.54 ±9.54
-1.14 ±0.02	-14.19 ±3.92	22.17 ±4.04	3.26 ±2.19	40.23 ±6.92	35.19 ±8.67	0.02	0.09	100.85 ±3.97
-1.23 ±0.02	-21.82 ±4.96	23.43 ±17.29	1.15 ±1.04	43.42 ±2.38	39.61 ±10.77	0.01	0.05	107.60 ±3.72
-1.31 ±0.02	-26.06 ±1.17	18.73 ±3.11	1.86 ±0.23	62.50 ±2.66	23.02 ±9.62	0.01	0.04	106.12 ±8.25

Table S3. Electrochemical CORR and CO_2RR studies of producing C_2 products on grain-boundary (GB)-rich and step/defect-rich Cu catalysts.

Catalyst	Reaction	Catalysis condition	Main Products	Reference
GB-rich Cu Nanoparticles	CORR	0.1 M KOH, H-cell	36% $FE_{C_2H_5OH}$, 34% FE_{OAc^-}, 28% FE_{H_2} at −0.3 V vs. RHE	16
Oxide-derived GB-rich Cu			43% $FE_{C_2H_5OH}$, 43% FE_{H_2} at -0.3 V vs. RHE	17
Cu Nanoparticles			> 94% FE_{H_2} at -0.3 to -0.5 V vs. RHE	
Oxide-derived GB-rich Cu			55% FE_{H_2}, 26% $FE_{C_2H_5OH}$, 14% FE_{OAc^-}, at -0.3 V vs. RHE	18
GB-rich Cu Nanoparticles	CO_2RR	1 M KOH, flow cell	38% $FE_{C_2H_4}$, 30% $FE_{C_2H_5OH}$, 12% FE_{H_2}, 8% FE_{CO} at -1.2 V vs. RHE; 35% $FE_{C_2H_4}$, 32% $FE_{C_2H_5OH}$, 14% FE_{H_2}, 4% FE_{CO} at −1.3 V vs. RHE	19
Oxide-derived GB-rich Cu		0.1M $KHCO_3$, H-cell	50% FE_{H_2}, 30% $FE_{C_2H_4}$, 10% FE_{CO}, 7% $FE_{C_2H_5OH}$ at -1.2 V vs. RHE	20
Step/Defect-rich Cu		0.1M $KHCO_3$, H-cell	53% $FE_{C_2H_5OH}$, 18% FE_{PrOH}, 13% FE_{H_2}, 7% $FE_{C_2H_4}$ at -1.1 V vs. RHE	21
		1 M KOH, flow cell	52% $FE_{C_2H_5OH}$, 14% FE_{PrOH}, 11% FE_{HCOO^-}, 8% FE_{H_2}, 7% $FE_{C_2H_4}$ at -1 V vs. RHE	

Table S4. Constant-charge DFT-PBE-D3 and approximated emb-CASPT2 activation free energies (G_{act}) and reaction free energies (ΔG) in eV for CO reduction via PCET to form *CHO and *COH and C-C coupling pathways to form *COH-CHO and *COH-*COH on tw-Cu(111).

	**DFT-PBE-D3**		**emb-CASPT2**	
	G_{act} (eV)	ΔG (eV)	G_{act} (eV)	ΔG (eV)
***CHO**	0.55	0.34	0.77	0.60
***COH**	0.21	0.21	0.92	0.92
***COH-CHO**	0.47	-0.96	0.44	-1.32
***COH-COH**	0.20	-1.28	0.42	-1.20

Table S5. Constant-charge DFT-PBE-D3 and emb-CASPT2 activation free energies (G_{act}) and reaction free energies (ΔG) in eV for CO reduction to form *CHO and *COH and C-C coupling pathways to form *COH-CHO and *COH-*COH on planar Cu(111)[a], as well as free energy differences between two levels of theory.

	**DFT-PBE-D3**		**emb-CASPT2**			
	G_{act} (eV)	ΔG (eV)	G_{act} (eV)	ΔG (eV)	$\Delta G_{act}^{ECW-DFT}$	$\Delta\Delta G^{ECW-DFT}$
***CHO**	0.85	0.53	1.07	0.79	0.22	0.26
***COH**	0.44	0.44	1.15	1.15	0.71	0.71
***COH-CHO**	0.34	-1.10	0.31	-1.46	-0.03	-0.36
***COH-*COH**	0.24	-1.32	0.46	-1.24	0.22	0.08

[a]The energetics for CO reduction via PCET to form *CHO and *COH and for C-C coupling pathways to form *COH-CHO and *COH-*COH on planar Cu(111) were taken respectively from Refs. 3 and 4.

Table S6. Adsorbate Bader charge changes between transition- and reactant-state structures ($\Delta q_{\text{TS-R}}$), and between product- and reactant-state structures ($\Delta q_{\text{P-R}}$) computed using embedded cluster models at the emb-PBE and emb-CASSCF level for CO reduction to form *CHO and *COH and C-C coupling pathways to form *COH-CHO and *COH-*COH on planar Cu(111), as well as adsorbate Bader charge change differences between the two levels of theory.

	**emb-PBE**		**emb-CASSCF**			
	$\Delta q_{\text{TS-R}}$	$\Delta q_{\text{P-R}}$	$\Delta q_{\text{TS-R}}$	$\Delta q_{\text{P-R}}$	$\Delta\Delta q_{\text{TS-R}}^{\text{ECW-DFT}}$	$\Delta\Delta q_{\text{P-R}}^{\text{ECW-DFT}}$
***CHO**	0.35	-0.03	0.21	-0.20	-0.14	-0.17
***COH**	0.31	0.31	-0.05	-0.05	-0.36	-0.36
***COH-CHO**	0.03	0.14	0.00	0.03	-0.03	-0.11
***COH-COH**	0.14	0.17	0.08	-0.00	-0.06	-0.18

[a]The structures and electron densities for CO reduction via PCET to form *CHO and *COH and for C-C coupling pathways to form *COH-CHO and *COH-*COH on planar Cu(111) were taken respectively from Refs. 3 and 4.

Table S7. Adsorbate Bader charge changes between transition- and reactant-state ($\Delta q_{TS\text{-}R}$), and product- and reactant-state ($\Delta q_{P\text{-}R}$) structures computed using periodic slab models at the DFT-PBE-D3 level for CO reduction via PCET to form *CHO and *COH and C-C coupling pathways to form *COH-CHO and *COH-*COH on planar Cu(111) and tw-Cu(111). Note that Δq on the two surfaces (planar and tw) agree to ~ 0.10 e for the same type of reaction.

	**planar Cu(111)**		**tw-Cu(111)**	
	$\Delta q_{TS\text{-}R}$	$\Delta q_{P\text{-}R}$	$\Delta q_{TS\text{-}R}$	$\Delta q_{P\text{-}R}$
***CHO**	0.34	-0.12	0.23	-0.03
***COH**	0.31	0.31	0.28	0.28
***COH-CHO**	0.10	0.07	0.09	0.05
***COH-*COH**	0.01	0.14	0.01	0.15

[a]The structures and electron densities for CO reduction via PCET to form *CHO and *COH and C-C coupling pathways to form *COH-CHO and *COH-*COH on planar Cu(111) were taken respectively from Refs. 3 and 4.

Table S8. Surface Bader charge (*q*, calculated from the tw-Cu(111) slab plus all of the adsorbates) and work function of the tw-Cu(111) slab with respect to the energy level of the bulk solvent in eV ($\Delta\Phi$), computed at the DFT-PBE-D3 level in the presence of implicit continuum solvation, and reaction (ΔG) and activation (G_{act}) free energies at constant $\Delta\Phi$ in eV at the DFT-PBE-D3 and emb-CASPT2 levels of theory for CO reduction to form *CHO and *COH via PCET on tw-Cu(111).

		q	**$\Delta\Phi$**	**DFT G_{act} or ΔG**		**ECW G_{act} or ΔG**	
				$\Delta\Phi_R$	$\Delta\Phi_P/\Delta\Phi_{TS}$	$\Delta\Phi_R$	$\Delta\Phi_P/\Delta\Phi_{TS}$
***CHO**	Reactant	-0.91	3.96	-	-	-	-
	TS	-0.10	4.23	0.44	0.66	0.65	0.88
	Product	-0.01	4.25	0.21	0.47	0.47	0.73
***COH**	Reactant	-0.91	3.96	-	-	-	-
	TS	-	-	-	-	-	-
	Product	-0.07	4.16	0.12	0.29	0.84	1.01

Table S9. Activation (G_{act}) and reaction (ΔG) free energies in eV for CO reduction to form *CHO and *COH via PCET at an applied potential of -1.2 V vs. RHE evaluated by emb-CASPT2 on tw-Cu(111) and planar Cu(111)[a].

		**tw-Cu(111)**		**planar Cu(111)**	
		G_{act} (eV)	ΔG (eV)	G_{act} (eV)	ΔG (eV)
**U = -1.2 V vs. RHE**	*CHO	0.00	-0.28	0.36	-0.02
	*COH	0.12	0.12	0.37	0.37

[a]The emb-CASPT2 energies, surface Bader charges, and work functions with respect to the energy level of the bulk solvent used to simulate potential-dependent activation and reaction free energies on planar Cu(111) were taken from Ref. 3. Unlike in Ref. 3, here, we report the energetics at more negative applied potentials of -1.2 V vs. RHE to compare the results between the planar Cu(111) and tw-Cu(111) directly with experiment (an applied potential of -0.9 V vs. RHE was used in the original work).

Table S10. DFT-PBE-D3 relative energies in eV for screened adsorption sites of each adsorbate on tw-Cu(111), referenced against the preferred adsorption sites. See Figure S7 for structures.

	**atop**	**bridge**	**hcp hollow**	**fcc hollow**	**bidentate atop**
***CO**	0.03	0.00	0.06	0.05	
***CHO**	0.24	0.17	0.17	0.17	0.00
***COH**	1.41	0.73	0.02	0.00	
	bridge	atop			
***COH-CHO**	0.00	0.20			
	fcc-hcp	atop-atop			
***COH-*COH**	0.00	0.71			

Reference

(1) K. Mathew, R. Sundararaman, K. Letchworth-Weaver, T. Arias, R. G. Hennig, Implicit Solvation Model for Density-Functional Study of Nanocrystal Surfaces and Reaction Pathways. *J. Chem. Phys.* **140**, 084106 (2014).

(2) K. Mathew, V. C. Kolluru, S. Mula, S. N. Steinmann, R. G. Hennig, Implicit Self-Consistent Electrolyte Model in Plane-Wave Density-Functional Theory. *J. Chem. Phys.* **151**, 234101 (2019).

(3) Q. Zhao, J. M. P. Martirez, E. A. Carter, Revisiting Understanding of Electrochemical CO_2 Reduction on Cu (111): Competing Proton-Coupled Electron Transfer Reaction Mechanisms Revealed by Embedded Correlated Wavefunction Theory. *J. Am. Chem. Soc.* **143**, 6152-6164 (2021).

(4) Q. Zhao, J. M. P. Martirez, E. A. Carter, Charting C-C Coupling Pathways in Electrochemical CO_2 Reduction on Cu(111) Using Embedded Correlated Wavefunction Theory. *Proc. Natl. Acad. Sci. U. S. A.* **119**, e2202931119 (2022).

(5) S. Xu, E. A. Carter, Optimal Functionalization of a Molecular Electrocatalyst for Hydride Transfer. *Proceedings of the National Academy of Sciences* **116**, 22953-22958 (2019).

(6) S. Xu, E. A. Carter, Balancing Competing Reactions in Hydride Transfer Catalysis Via Catalyst Surface Doping: The Ionization Energy Descriptor. *J. Am. Chem. Soc.* **141**, 9895-9901 (2019).

(7) A. J. Cohen, P. Mori-Sánchez, W. Yang, Insights Into Current Limitations of Density Functional Theory. *Science* **321**, 792-794 (2008).

(8) R. F. Bader, A Quantum Theory of Molecular Structure and Its Applications. *Chem. Rev.* **91**, 893-928 (1991).

(9) W. Tang, E. Sanville, G. Henkelman, A Grid-Based Bader Analysis Algorithm Without Lattice Bias. *J. Phys. Condens. Matter.* **21**, 084204 (2009).

(10) B. O. Roos, The Complete Active Space SCF Method in a Fock-Matrix-Based Super-CI Formulation. *Int. J. Quantum Chem.* **18**, 175-189 (1980).

(11) P. E. M. Siegbahn, J. Almlöf, A. Heiberg, B. O. Roos, The Complete Active Space SCF (CASSCF) Method in a Newton–Raphson Formulation with Application to the HNO Molecule. *J. Chem. Phys.* **74**, 2384 (1981).

(12) K. Chan, J. K. Nørskov, Electrochemical Barriers Made Simple. *J. Phys. Chem. Lett.* **6**, 2663-2668 (2015).

(13) K. Chan, J. K. Nørskov, Potential Dependence of Electrochemical Barriers from Ab Initio Calculations. *J. Phys. Chem. Lett.* **7**, 1686-1690 (2016).

(14) J. K. Nørskov, J. Rossmeisl, A. Logadottir, L. Lindqvist, J. R. Kitchin, T. Bligaard, H. Jónsson, Origin of the Overpotential for Oxygen Reduction at a Fuel-Cell Cathode. J. Phys. Chem. B. 108, 17886-17892 (2004).

(15) L. Li, J. M. P. Martirez, E. A. Carter, Prediction of Highly Selective Electrocatalytic Nitrogen Reduction at Low Overpotential on a Mo-doped G-GaN Monolayer. *ACS Catal.* **10**, 12841-12857 (2020).
(16) X. Feng, K. Jiang, S. Fan, M. W. Kanan, A Direct Grain-Boundary-Activity Correlation for CO Electroreduction on Cu Nanoparticles. *ACS Cent. Sci.* 2, 169-174 (2016).
(17) C. W. Li, J. Ciston, M. W. Kanan, Electroreduction of Carbon Monoxide to Liquid Fuel on Oxide-Derived Nanocrystalline Copper. *Nature.* 508, 504-507 (2014).
(18) A. Verdaguer-Casadevall, C. W. Li, T. P. Johansson, S. B. Scott, J. T. McKeown, M. Kumar, I. E. L. Stephens, M. W. Kanan, I. Chorkendorff, Probing the Active Surface Sites for CO Reduction on Oxide-Derived Copper Electrocatalysts. *J. Am. Chem. Soc.* 137, 9808-9811 (2015).
(19) Z. Chen, T. Wang, B. Liu, D. Cheng, C. Hu, G. Zhang, W. Zhu, H. Wang, Z.-J. Zhao, J. Gong, Grain-Boundary-Rich Copper for Efficient Solar-Driven Electrochemical CO_2 Reduction to Ethylene and Ethanol. *J. Am. Chem. Soc.* 142, 6878-6883 (2020).
(20) D. Cheng, Z.-J. Zhao, G. Zhang, P. Yang, L. Li, H. Gao, S. Liu, X. Chang, S. Chen, T. Wang, G. A. Ozin, Z. Liu, J. Gong, The Nature of Active Sites for Carbon Dioxide Electroreduction over Oxide-Derived Copper Catalysts. *Nat Commun.* 12, 395 (2021).
(21) Z. Gu, H. Shen, Z. Chen, Y. Yang, C. Yang, Y. Ji, Y. Wang, C. Zhu, J. Liu, J. Li, Efficient Electrocatalytic CO_2 Reduction to C_{2+} Alcohols at Defect-Site-Rich Cu Surface. *Joule.* 5, 429-440 (2021).
